# Implementation of the world health organization hand hygiene improvement strategy in critical care units

**DOI:** 10.1186/2047-2994-2-15

**Published:** 2013-05-14

**Authors:** Waleed Mazi, Abiola C Senok, Sameera Al-Kahldy, Diaa Abdullah

**Affiliations:** 1 Infection Prevention and Control Department, King Abdul Aziz Specialist Hospital, Taif, Saudi Arabia; 2 Department of Pathology and Pharmacology, College of Medicine, Alfaisal University, Riyadh, Saudi Arabia

**Keywords:** Hand hygiene, Critical care, Infection control, WHO strategy

## Abstract

**Background:**

To determine hand hygiene compliance before and after an intervention campaign in critical care units, this study was carried out in the Intensive care unit (ICU), Neonatal intensive care unit (NICU), Burns unit (BU) and the Kidney unit of the King Abdul Aziz Specialist Hospital, Taif, Saudi Arabia. The observation using the WHO hand hygiene protocol took place in four phases with phase I, between April 24-May 06 2010 and phase II from May 29-June 09 2010. An educational intervention took place between the Phases I and II. Follow-up Phases III and IV were from 01–15 October 2010 and 15–30 March 2011 respectively.

**Findings:**

1,975 hand hygiene opportunities comprising of 409 in Phase I, 406 in Phase II, 620 in Phase III and 540 Phase IV were observed. Compliance rate was 67% pre-intervention, 81% in phase II, declining to 59% and 65% in phases III and IV. Increased compliance in the ICU from 39% in Phase I to 81% in Phase IV (p < 0.05) was sustained throughout the study. Highest compliance rates were recorded among nurses in all phases. The improved compliance for physicians observed in the post-intervention phase was lost in follow-up phases. Missed opportunities for hand hygiene were before patient contact, after touching patient’s surrounding and before aseptic techniques. Team-work and leadership were identified as enhancing factors for compliance.

**Conclusion:**

The WHO hand hygiene strategy combined with health education, continuous evaluation and team approach resulted in increased compliance but this was not sustained in certain critical care areas.

## Introduction

The importance of hand hygiene in infection control was first demonstrated over 150 years ago by Ignaz Semmelweis [1]. The practice of hand hygiene is now recommended as a core element of infection prevention and control for patient safety. This is particularly pertinent in view of the global emergence and rapid dissemination of multidrug resistant pathogens which is associated with increased patient morbidity and mortality [2]. However, despite the fact that hand hygiene has been demonstrated as the single most important strategy to prevent and control healthcare associated infections and spread of multi-drug resistance organisms in healthcare settings, studies have shown that hand hygiene compliance remains suboptimal [2-4].

The World Health Organization launched the First Global Patient Safety Challenge - Clean Care is Safer Care - in 2005 which has as its goal the global reduction of HAI. In 2009, the SAVE LIVES: Clean Your Hands initiative was launched to emphasize the importance of hand hygiene in HAI prevention [5]. To standardize the best implementation of hand hygiene practices, a user-centered approach to understand, train, monitor and report hand hygiene compliance was introduced [6]. This concept known as “My five moments for hand hygiene” describes the fundamental reference point for health care personnel in a time-space framework and designates when hand hygiene is required for effective interruption of microbial transmission during patient care [6,7]. It is applicable to a wider variety of patient care activities and healthcare settings with the five moments being: Before patient contact (Moment 1); Before an aseptic task (Moment 2); After body fluid exposure risk (Moment 3); After patient contact (Moment 4); After contact with patient surroundings (Moment 5) [5,7]. It provides a standardized tool that facilitates education, implementation and monitoring of adherence to hand hygiene practices. King Abdul Aziz Specialist Hospital (KAASH), Taif, Saudi Arabia became a participating hospital in World Health Organization (WHO) SAVE LIVES: Clean Your Hands Initiative in 2010. This study was designed to determine hand hygiene compliance using the WHO global hand hygiene observation protocol, before and after an intervention campaign. Adherence to hand hygiene practices and identification of appropriate strategies for ensuring compliance was addressed.

## Methods

KAASH is a 545-bed tertiary care facility with inpatient and outpatient specialized healthcare services being delivered in different clinical departments. Hand hygiene compliance was assessed using the WHO Hand Hygiene Observation Form in four critical care units namely: Intensive Care Unit (ICU), Neonatal ICU (NICU), Kidney center and Burns Unit (BU). Observation of hand hygiene compliance was carried out by the “Hand Hygiene Observation Team (HHOT) which is comprised of trained members of Infection Prevention and Control Department (IPCD). The observation took place in the relevant departments in four phases with phase 1 or pre-intervention between April 24-May 06 2010 and phase 2 or post-intervention phase from May 29-June 09 2010. Phases 3 and 4 were sequential follow up phases from 01–15 October 2010 and 15–30 March 2011 respectively. During each phase, observers visited the hospital units at designated times. The staff in each unit was aware that observation of hand hygiene compliance was being conducted during the designated times.

The improvement tools used included an educational program in the form of a hand hygiene intervention campaign which took place between Phases 1 and 2. The program was in the form of lectures and hands-on workshop. Importance of hand hygiene, strategies for changing behaviors, hand hygiene practices, methods of hand hygiene observation were addressed. Hands-on workshop and scientific exhibition to demonstrate ‘my five moments for hand hygiene’, appropriate method for hand hygiene and latest products for hand hygiene was conducted. The head nurse in each unit was the team leader for the implementation of hand hygiene compliance in their unit. The IPCD provided quarterly feedback reports to the team leaders and the hospital director. Regular open discussion on compliance rates were held during the hospital infection control committee meetings convened by the IPCD. As part of this process, data on factors which enhanced compliance and those perceived as barriers to compliance were collected. Throughout the study duration, regular supply of alcohol gel/chlorhexidine was maintained in all units.

Statistical analysis was performed using SigmaStat ver 3.5 software (Systat Software Inc, San Jose California, USA) and *p* < 0.05 was considered statistically significant.

Ethical approval for this study was obtained from the King Abdul Aziz Specialist Hospital, Taif.

## Results

A total of 1,975 hand hygiene opportunities were observed comprising of 409 in Phase 1, 406 in Phase II with 620 and 544 in Phases III and Phase IV respectively. The overall hospital wide hand hygiene compliance rate was 67% in the pre-intervention phase, increased to 81% in the post-intervention phase, but declined to 59% and 65% in Phases III and IV respectively (Table 1). In the pre-intervention phase, the highest compliance rate was in the NICU (88%) and lowest in the kidney center (36%) (Table 1). High levels (≥88%) of compliance was maintained across the study period in the NICU except for Phase IV when 68% compliance was recorded. Significant sustained improvement in hand hygiene compliance was observed in the ICU throughout the study period increasing from 39% in Phase I to 81% in Phase IV (p < 0.05) (Table 1). In contrast, significant decline in compliance in the burns unit across the study periods (70% pre-intervention to 53% in phase IV) was observed. In the kidney unit, the improved compliance observed in the post-intervention period, was statistically significant (pre: 43.37%; post: 70.8%, *p* < 0.001).

**Table 1:** Distribution of hand hygiene compliance across critical care units in the hospital

**Critical care units**	**Percentage of hand hygiene compliance (# of actions/opportunities for hand hygiene)**			
	**Phase I**	**Phase II**	**Phase II**	**Phase IV**
Intensive care unit (ICU)	39 (21/54)	57 (29/51)	53 (182/344)	81 (130/160)
Neonatal intensive care unit (NICU)	88 (142/162)	90 (145/160)	90 (94/104)	68 (177/260)
Burn Unit	70 (77/110)	85 (93/110)	78 (46/59)	53 (32/100)
Kidney Center	43 (36/83)	71 (60/85)	36 (41/113)	54 (13/24)
***Total***	***67 (276/409)***	***81 (327/406)***	***59 (363/620)***	***65 (379/540)***

The compliance among nurses went from 63% pre-intervention to 76% in Phase IV. In Phase IV, higher compliance rates were maintained by nurses in the ICU, NICU and BU relative to other categories of healthcare personnel (Table 2). In contrast, the improved compliance for physicians observed between the pre-intervention and post-intervention periods was lost by Phases III & IV (Table 2). In the kidney unit, opportunities for hand hygiene were not observed among physicians because most of the practices for dialysis were carried out by nurses. The main sources of missed action were in order of decreasing magnitude: Moment 1: Before patient contact (with/without donning gloves); Moment 5: after touching patient surrounding and Moment 2: before aseptic techniques when they don gloves directly without hand hygiene.

**Table 2:** Distribution of hand hygiene compliance among healthcare professionals in critical care units in the hospital

**Critical care unit**	**Hand hygiene compliance (%)**											
	**Phase I**			**Phase II**			**Phase III**			**Phase IV**		
	**Nurses**	**Physicians**	**Others**	**Nurses**	**Physicians**	**Others**	**Nurses**	**Physicians**	**Others**	**Nurses**	**Physicians**	**Others**
ICU	45	41	29	45	63	45	55	49	54	87	69	73
NICU	90	78	98	88	90	-	90	84	97	80	68	50
Burn Unit	71	50	-	80	50	-	78	-	-	75	48	6
Kidney Center	42	-	66	70	-	-	40	0	17	53	-	50
Compliance rate% (*)	***63 (142/225)***	***56 (61/108)***	***65 (50/76)***	***73 (183/250)***	***78 (71/90)***	***45 (30/66)***	***61 (229/373)***	***52 (71/137)***	***57 (63/110)***	***76 (235/309)***	***59 (93/158)***	***55 (40/73)***

*Number of actions/opportunities for hand hygiene.

The hand hygiene campaign resulted in enhanced hand hygiene in all the units in the post-intervention period. In Phases III and IV, team effort and having a team leader was identified as a significant positive factor in the ICU and NICU. A barrier to hand hygiene compliance reported from all units related to the quality and placement of the alcohol handrub. In particular, in the kidney and burns unit, placement of the alcohol hand rubs away from the patients was perceived as not being strategic for adequate compliance.

## Discussion

The WHO “my five moments for hand hygiene” represents a standardized approach for training, implementation, monitoring and reporting of hand hygiene compliance. The five moments identified in this strategy are: Moment 1: Before patient contact; Moment 2: Before an aseptic task; Moment 3: After body fluid exposure risk; Moment 4: After patient contact and Moment 5: After contact with patient surroundings [5,7]. Although optimal compliance with hand hygiene remains a cornerstone of preventing HAIs, studies in developed and developing countries continue to demonstrate sub-optimal compliance. A myriad of factors are associated with poor compliance with significant variations in monitoring and reporting according to the setting and resources available. Identification of factors and specific timings missed opportunities during patient care is critical so that these can be addressed for future compliance strategies. In contrast to previous reports from Saudi Arabia, this is the first study documenting the utilization of the WHO hand hygiene observation method in the country [8-10]. With this approach, the key moments of missed opportunity during patient care our critical care units were identified as Moments 1, 2 and 5. The WHO hand hygiene observation method provides objective identification of the missed opportunities for hand hygiene which can be targeted in future educational campaigns.

To improve adherence to hand hygiene practices, several interventions such as performance feedback on hand hygiene compliance, display of hand hygiene posters and introduction of alcohol-based hand rubs have been described.[11,12]. A similar multimodal approach has been incorporated in this study, and an extended follow-up of the intervention strategy was conducted [11]. In addition, we have evaluated the long term impact of educational campaign in the implementation of this hand hygiene strategy. Similar to other studies, the findings indicate a poor baseline hand hygiene compliance with significant improvement observed in the immediate post intervention period [13,14]. However, extended follow up shows that this high level of improved compliance was not sustained as shown by decline in Phases III and IV. It is however notable that at the end of the study period, overall improvement in hand hygiene compliance was observed. The observed inability to achieve sustained hand hygiene compliance suggests that changing behavior is complex. Intervention campaigns such as that carried out in this study play a role in temporary behavior modification; however, for sustained hand hygiene compliance other factors are indicated. Various researchers have applied the theory of planned behavior to investigate hand hygiene compliance [15,16]. One of the important postulates of this theory is that intention to perform hand hygiene is influenced by beliefs about the expectations of others which are perceived as important [15,16]. The effect of this form of social pressure in behavior modification is not uniformly reflected in our findings. As the reporting of results of hand hygiene observation to healthcare workers is an essential element of multi-modal strategies to improve hand hygiene practices, regular feedback and open discussions were carried out. However, this did not have a universal effect of improving compliance rates in all hospital units and across all categories of staff. This approach was found to be effective in the NICU and ICU where the trend of increased compliance was attributed to teamwork and having a team leader. A team approach with the guidance of a team leader has been suggested as a modality for behavior change in sustaining hand hygiene compliance [17,18]. Further study to identify effective parameters and explore the role of team leaders for sustained behavior modification in our setting is being undertaken.

High levels of compliance were found among nursing staff. This is similar to other reports in the literature [19]. However, similar to other reports physician compliance remained very low and the absence of a physician leader was identified as a barrier to improving compliance among doctors in all units. [19,20]. Although institutional backing and individual education are critical for the success of the hand hygiene implementation program, our findings show that these strategies are by themselves insufficient for sustaining compliance. A recent study has shown that repeated hand hygiene campaigns over a one year period only marginally increased compliance [9]. The involvement of physicians and nursing staff managers in hand hygiene activities, dissemination of feedback and evaluations has been suggested as critical [17,18,20]. Indeed, the presence of team leaders contributed to increased compliance in among the nursing staff in general. However, lack of physician team leader was detrimental to compliance rates among physicians.

## Conclusion

In conclusion, we have shown that a multi-pronged approach of health education campaign, continuous evaluation and feedback as well as incorporation of team work and team leaders have led to increased compliance in hand hygiene in our setting. The utilization of the WHO ‘my five moments for hand hygiene” strategy identified the key moments of missed opportunities during patient care. Further studies are needed to identify strategies for continued long term maintenance of hand hygiene compliance.

## Abbreviations

BU: Burns unit; HHOT: Hand hygiene observation team; ICU: Intensive care unit; IPCD: Infection prevention and control department; KAASH: King Abdul Aziz specialist hospital; NICU: Neonatal ICU; WHO: World health organization.

## Competing interests

The authors (Waleed Mazi; Abiola Senok; Sameera Al- Kahldy and Diaa Abdullah) declare that they have no personal or financial relationship which may constitute a conflict of interest.

## Authors’ contributions

All authors have made substantial intellectual contribution to this work as follows: WM: Conception and design of the study, data acquisition and analysis as well preparation of the manuscript draft and critical revision of final manuscript. AS: Conception and design of study, data analysis and interpretation, preparation of manuscript draft and critical revision of manuscript. SK: Study design, Data acquisition and critical revision of manuscript. DA: Study design, Data acquisition and critical revision of manuscript. All authors read and approved the final manuscript.
